# Self-association of human beta-galactocerebrosidase: Dependence on pH, salt, and surfactant

**DOI:** 10.1371/journal.pone.0226618

**Published:** 2019-12-23

**Authors:** Eunhee Lee, Nazila Salamat-Miller, Walter F. Stafford, Katherine Taylor

**Affiliations:** 1 Process Development and Technical Services, Shire US Inc., a Takeda company, Lexington, MA, United States of America; 2 Cardiovascular Biology Program and AUC Research Laboratory, Boston Biomedical Research Institute, Watertown, MA, United States of America; Graduate School of Agriculture, Kyoto University, JAPAN

## Abstract

Krabbe disease, also known as globoid cell leukodystrophy, is a rare genetic neurodegenerative disease caused by a deficiency of the galactocerebrosidase enzyme. To understand the association status of human beta-galactocerebrosidase (hGALC) in solution, we employed analytical ultracentrifugation (AUC). Our AUC results show that hGALC has a tendency for reversible self-association. Self-association decreases as the concentration of sodium chloride increases from 50 to 500 mM. This indicates that ionic interactions are involved in the association. The association is also dependent on pH, and high order oligomerization decreases as the pH increases from 4.5 to 7.5. Taken together, our results indicate that hGALC has the highest tendency for oligomerization at physiological ionic strength and pH (lysosomal lumen). This is the first report describing the self-associating property of hGALC in solution.

## Introduction

Krabbe disease (aka Globoid cell leukodystrophy) is a lysosomal storage disease and inherited as an autosomal recessive disorder. It is a rare disease, and people with Krabbe disease have a deficiency of the galactocerebrosidase enzyme. The lysosomal galactocerebrosidase enzyme catalyzes the hydrolysis of galactose from glycosphingolipids, such as galactosylceramide and galactosylsphingosine. Abnormal metabolism of sphingolipids results in progressive damage to the nervous system. The human galactocerebrosidase enzyme (hGALC) was purified from brain and liver tissues forty years ago [1]. The authors reported that hGALC exists in multiple molecular weight (MW) forms based on results from gel filtration, SDS-PAGE and immunoprecipitation. In addition, they suggested that the high MW form (approx. 750 kDa) is active *in vivo* because it showed higher specific activity toward natural substrates than the low MW form (approx. 125 kDa). hGALC purified from other tissues also exhibited multiple MW forms [2–4]. Although the cDNA encoding hGALC was cloned [5–6], the status of hGALC self-association has nonetheless been unknown. We, therefore, characterized the solution behavior of recombinant hGALC using analytical ultracentrifugation. We assessed the effects of ionic strength, pH, and a cholate-based surfactant on self-association of hGALC. In addition, we discuss the implications of our findings in relation to the lysosome compartment.

## Materials and methods

### Sample preparation

The recombinant human galactocerebrosidase (hGALC) protein was provided by using cell culture and a purification process developed at Shire US Inc., a member of the Takeda group of companies, Lexington, MA. Briefly, hGALC was expressed in a human cell line HT1080, purchased from ATCC. The culture broth containing hGALC was clarified by centrifugation/filtration to remove cells and cell debris prior to chromatography. Purification was carried out using a series TOYOPEARL GigaCap Q-650M anion exchange, TOYOPEARL PPG-600M hydrophobic interaction, and POROS^TM^ 50HS cation exchange chromatography. TOYOPEARL GigaCap Q-650M and PPG-600M were purchased from Tosoh Bioscience LLC and POROS^TM^ 50HS from Thermo Fisher Scientific Inc. Subsequently, filtration with Planova^TM^ 20N (from Asahi Kasei Medical Co., Ltd) was performed to remove endogenous/adventitious viruses. Ultrafiltration/diafiltration was done using Pellicon® 3 cassette with an Ultracel® 30 kDa membrane (from MilliporeSigma) to concentrate and formulate hGALC in 5 mM sodium phosphate, pH 6.3 and 150 mM sodium chloride (NaCl). Chemicals for buffer preparation were purchased from J. T. Baker except for sodium taurocholate purchased from Sigma. To examine the effect of ionic strength on self-association, hGALC was dialyzed against buffers containing varying amounts of NaCl (5 mM sodium phosphate, pH 6.0 with 50–500 mM NaCl) for 24 hours at 4°C. To assess the association status at various pH levels, hGALC was dialyzed against buffers differing in pH (3 mM citrate, phosphate, and borate, and 50 mM NaCl, pH 4.5–7.5) for 24 hours at 4°C. To evaluate the effect of sodium taurocholate on self-association, hGALC was dialyzed against a buffer consisting of 5 mM sodium phosphate (pH 6.0), 150 mM NaCl and 1% sodium taurocholate for 24 hours at 4°C. Serial-dilutions were made immediately prior to each AUC run using the dialysate. To get the refractive index contribution of the protein, the dialysate was used as an optical reference. Although 24-hour dialysis was employed in this study, gel filtration (by ordinary column gel filtration or spin columns) can be used to achieve AUC samples in osmotic equilibrium with their respective buffers [7]. When gel filtration or spin columns are employed, the column buffer that was used to equilibrate the column must be employed for dilutions and as an optical blank. As the refractive index match between the sample buffer and the reference buffer must be exact, an aliquot of buffer of nominally the same composition will not match sufficiently well to act as reference. After matching the meniscus between the sample and the reference, temperature equilibration was done for 1 hour at 20°C.

### Analytical ultracentrifugation

Sedimentation velocity experiments were done on a Beckman Instrument Optima XL-I analytical ultracentrifuge equipped with Rayleigh optics. The cells were equipped with sapphire windows and 12 mm charcoal-filled Epon centerpieces. Sedimentation velocity runs were executed at 40,000 rpm and 20°C. Apparent sedimentation coefficient (s*) distribution patterns were computed by the time derivative method [8–10]. To examine the polydisperse hGALC system spanning a broad range of s*, the Wide Distribution Analysis (WDA) module in the SedAnal program [11] was used. Note that DCDT (i.e. g(s*)) is not a fit but a snapshot of the run at a specific time. WDA uses all the scans and avoids choosing specific scans. Using data taken at a set of radii 6.4–6.6, WDA plots s*g(s*) vs log_10_(s*) which are interpolated onto a log_10_(s*) grid before being smoothed or averaged. All WDA plots were corrected for the density, viscosity, and partial specific volume. Sednterp [12] was employed to compute values of density (ρ) and viscosity from the buffer composition (Table 1) and $\bar{v}$ from the amino acid sequence.

**Table 1:** Computed values of density and viscosity of buffers.

Buffer	Density (g/cm^3^)	Viscosity (cP)
50 mM NaCl^a^	1.0009	1.0080
150 mM NaCl^a^	1.0050	1.0169
500 mM NaCl^a^	1.0193	1.0494
pH^b^	1.0004	1.0071

^a^5 mM sodium phosphate, pH 6.0 with the designated concentration of NaCl.

^b^3 mM citrate, phosphate, and borate, and 50 mM NaCl, pH 4.5–7.5.

To consider the carbohydrate moiety of hGALC, the weight average $\bar{v}$ was calculated as follows [13]:

$$\bar{v}\left\{weight\;average\right\}=\frac{\left[M_{P}\;\text{x}\;{\bar{v}}_{P}\right]+\left[\left(M_{GP}-M_{P}\right)\;\text{x}\;{\bar{v}}_{G}\right]}{M_{GP}}$$

where *M*_*P*_ is the theoretical molecular mass (MW) of the protein moiety (73 kDa computed from the amino acid sequence), *M*_*GP*_ the measured MW of the glycoprotein (79 kDa measured with a mass spectrometry), ${\bar{v}}_{P}$ the partial specific volume of the protein moiety (0.738 cm^3^ g^-1^ computed from the amino acid sequence), and ${\bar{v}}_{G}$ the partial specific volume of the carbohydrate moiety (0.622 cm^3^ g^-1^, mean of a 0.602–0.642 range [13] due to unknown glycan structures). Molar mass was computed from sedimentation velocity profiles using the SedAnal program, which uses a nonlinear least-squares curve fitting algorithm to fit data to solutions of the differential equation (the Lamm equation) describing sedimentation. Fits were performed on time difference data to remove the time-independent systematic baseline components. Values of s and D produced by the fitting procedure were substituted into the Svedberg equation to obtain the molar mass of the protein, M:

$$\text{M}=\frac{\text{RT}}{\left(1-\bar{v}\text{ρ}\right)}\frac{{\text{s}}_{20,\text{w}}^{\text{o}}}{{\text{D}}_{20,\text{w}}^{\text{o}}}$$

where ρ is the density of the buffer and $\bar{v}$ the partial specific volume of the protein.

## Results

### Rationale for the analytical ultracentrifugation (AUC) study

AUC was employed to characterize the sedimentation coefficient of the purified recombinant hGALC because results from other analytical methods were incomprehensible. For instance, a size exclusion chromatography showed multiple unresolved peaks, and the peak profile changed upon varying pH and the salt content. Dynamic light scattering results indicated the presence of many species. Field-flow fractionation showed that MW and heterogeneity increased in proportion to the concentration.

### Self-association of hGALC was influenced by the ionic strength

**Fig 1:**
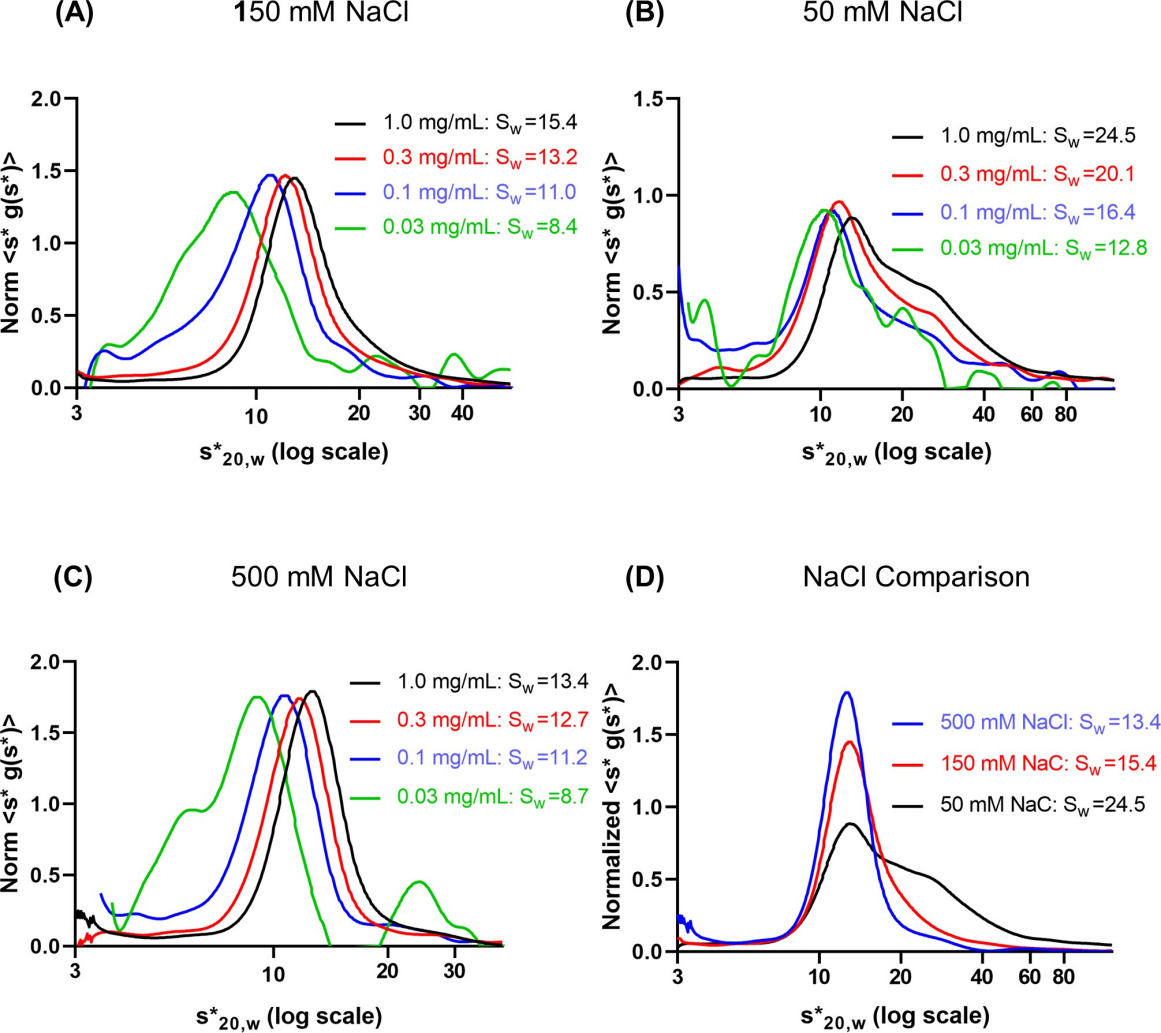
The NaCl effect on self-association of hGALC. Concentration-normalized s*g(s*) vs log_10_(s*) curves from 150 mM **(A)**, 50 mM **(B)** and 500 mM NaCl **(C)** in 5 mM sodium phosphate pH 6.0: The logarithmic s* scale was used in order to incorporate a wide range of s* in a single graph. The peak position shifted to lower s values upon dilution, indicating a decrease in self-association. The weight-average sedimentation coefficients (S_W_) were corrected to account for differences in the density and viscosity of the buffers. **(D)** 1 mg/mL from each of the ionic strength. Self-association/aggregation decreased as the ionic strength increased: 50 mM > 150 mM > 500 mM NaCl.

A sedimentation velocity dilution series was run on hGALC in 5 mM sodium phosphate, pH 6.0 with 150 mM sodium chloride (NaCl) to examine self-association and/or non-ideality of hGALC. The data were plotted as concentration-normalized s*g(s*) vs log_10_(s*) using the Wide Distribution Analysis (WDA) module in the SedAnal program to assess species spanning a broad range of apparent sedimentation coefficients (s*). The peak position moved to lower s values upon dilution, indicating reversible self-association/dissociation (Fig 1A).

This suggested that the equilibrium shifted toward smaller s species. In addition, the presence of higher aggregates (s* > 20 S) was evident. Subsequently, the association behavior was assessed in lower (50 mM) or higher (500 mM) NaCl concentration (Fig 1B and 1C). The results showed that higher degree of oligomerization at 50 mM NaCl and lower at 500 mM (Fig 1D). This indicated that ionic interactions were involved in self-association. There was a possibility that the self-association was heterogeneous due to the presence of incompetent species for reversible association/dissociation reactions. To determine the association status, fitting was performed using the SedAnal program. hGALC is a lysosomal enzyme and heavily glycosylated [14–16]. The glycan profile of hGALC was complex and the identity of each species was not determined. Therefore, a weight-average $\bar{v}$ was employed for fitting. We hesitate to estimate the s value of the largest oligomer since it is not highly populated. Although the association behavior under 500 mM NaCl appeared to be uncomplicated, the system was very heterogeneous and not at equilibrium in all four dilutions due to slow kinetics. In other words, the reaction was not complete at the concentrations used in the experiments. The tetramer had an f/f_0_ = 1.15 and s = 14.8 S from fits to a model of monomer-dimer-trimer-tetramer. Thus, the largest oligomer must be greater than 14.8 S because the fits suggested the presence some species larger than tetramer which were not accounted for the fits. In conclusion, the identity of the next higher oligomer was not determined due to the presence in such low amount. Therefore, its identity could not be determined with any degree of certainty.

### Higher pH shifted the equilibrium of self-association of hGALC to favor the smaller species

**Fig 2:**
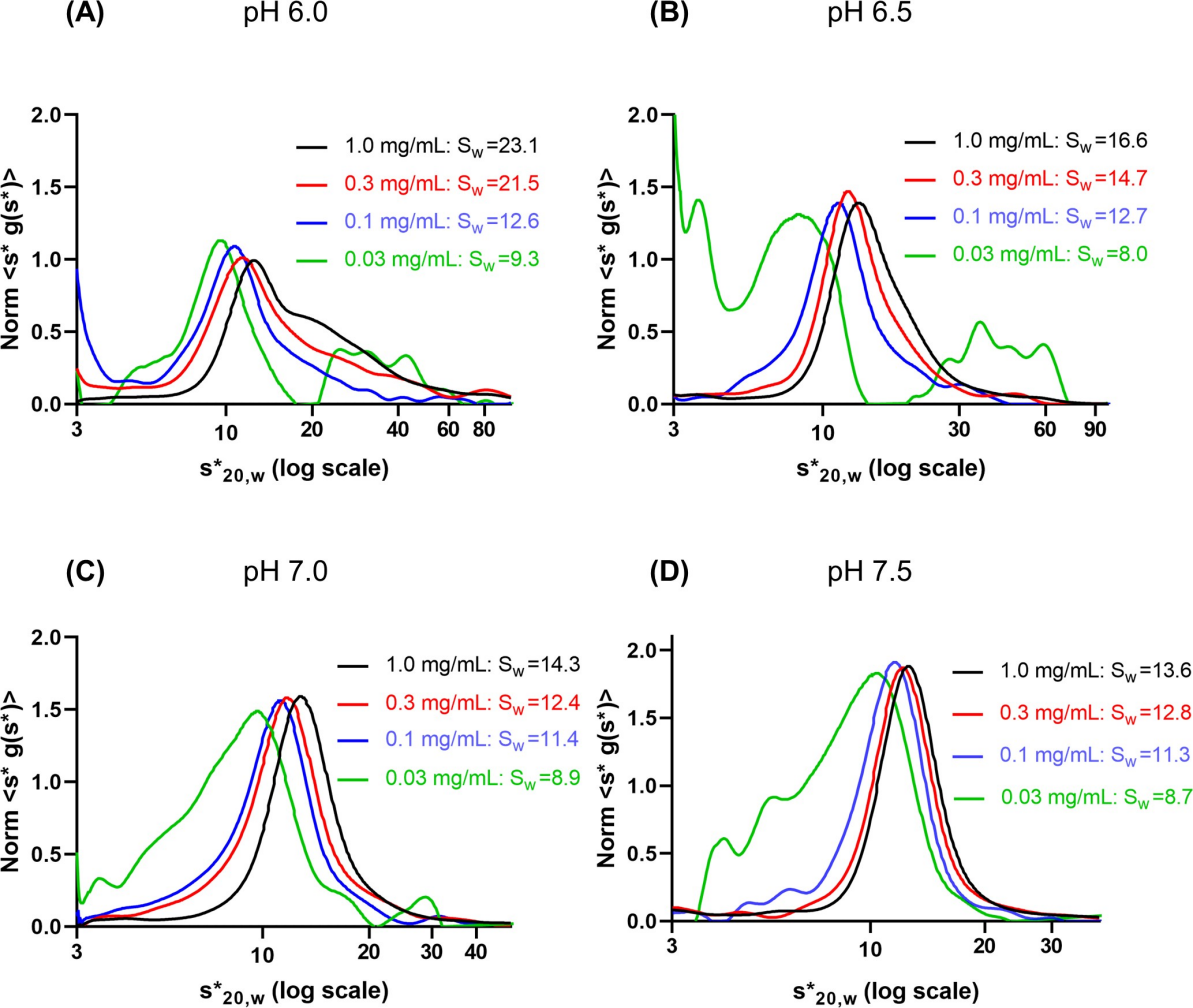
The pH effect on self-association of hGALC. Concentration-normalized s*g(s*) vs log_10_(s*) curves from pH 6.0 **(A)**, 6.5 **(B)**, 7.0 **(C)**, and 7.5 **(D)** in 50 mM NaCl: The peak position shifted to lower s values upon dilution, indicating decrease in self-association. S_W_ were corrected for the density and viscosity of the buffer.

To examine the pH effect on self-association, a sedimentation velocity dilution series was run on hGALC in 3 mM sodium citrate, phosphate, borate, and 50 mM NaCl at pH 4.5–7.5. The propensity for self-association decreased as the pH increased from pH 6.0 to 7.5 (Fig 2).

**Fig 3:**
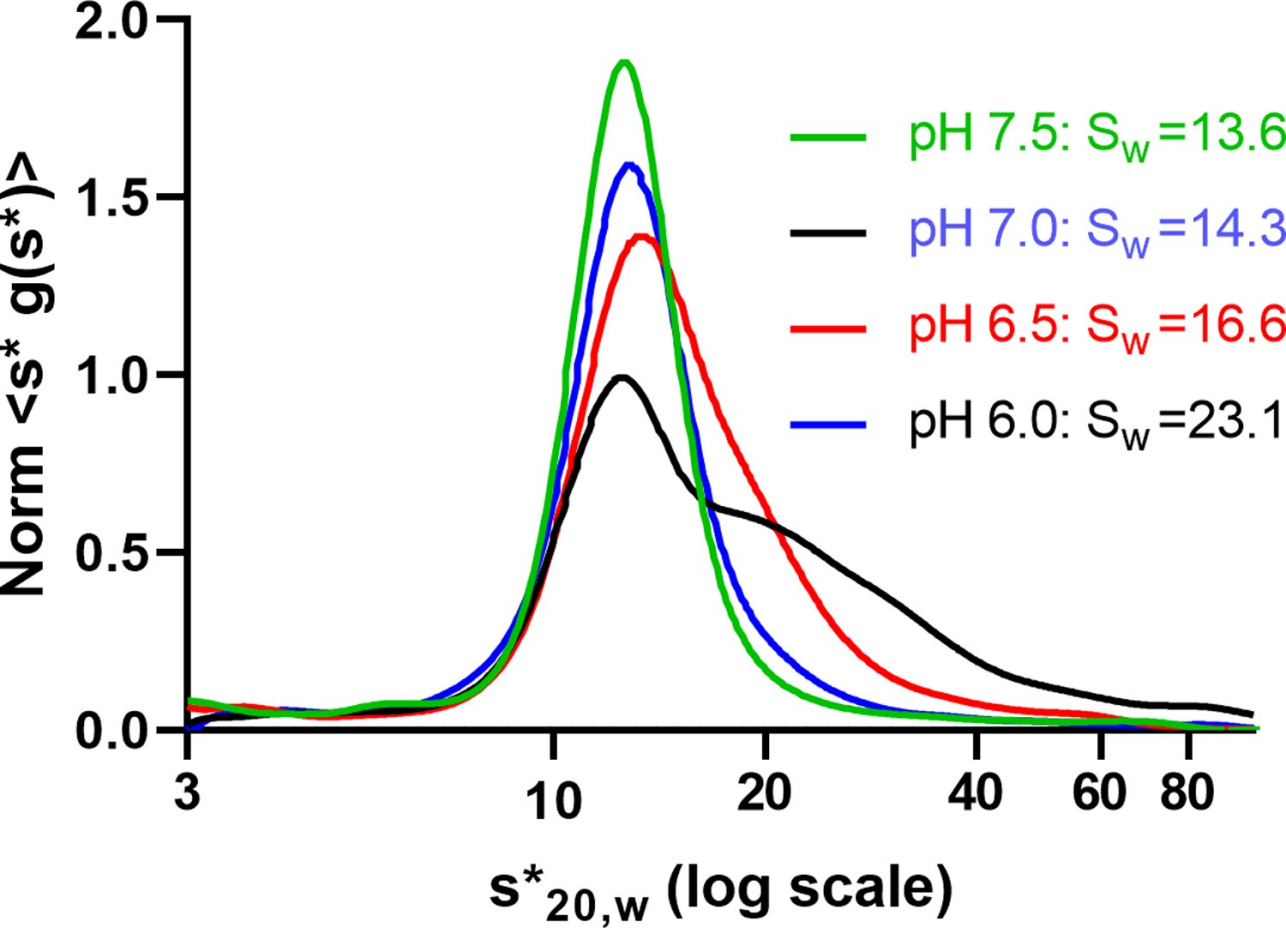
Summary of the pH-dependent oligomerization of hGALC. Concentration-normalized s*g(s*) vs log_10_(s*) curves of 1 mg/mL from each of pH levels: Self-association/ high-order oligomerization decreased as pH increased: pH 6.0 > 6.5 > 7.0 > 7.5. S_W_ were corrected for the density and viscosity of the buffer.

The pH-dependent self-association was manifest in the graph of 1 mg/mL from each sample (Fig 3).

**Fig 4:**
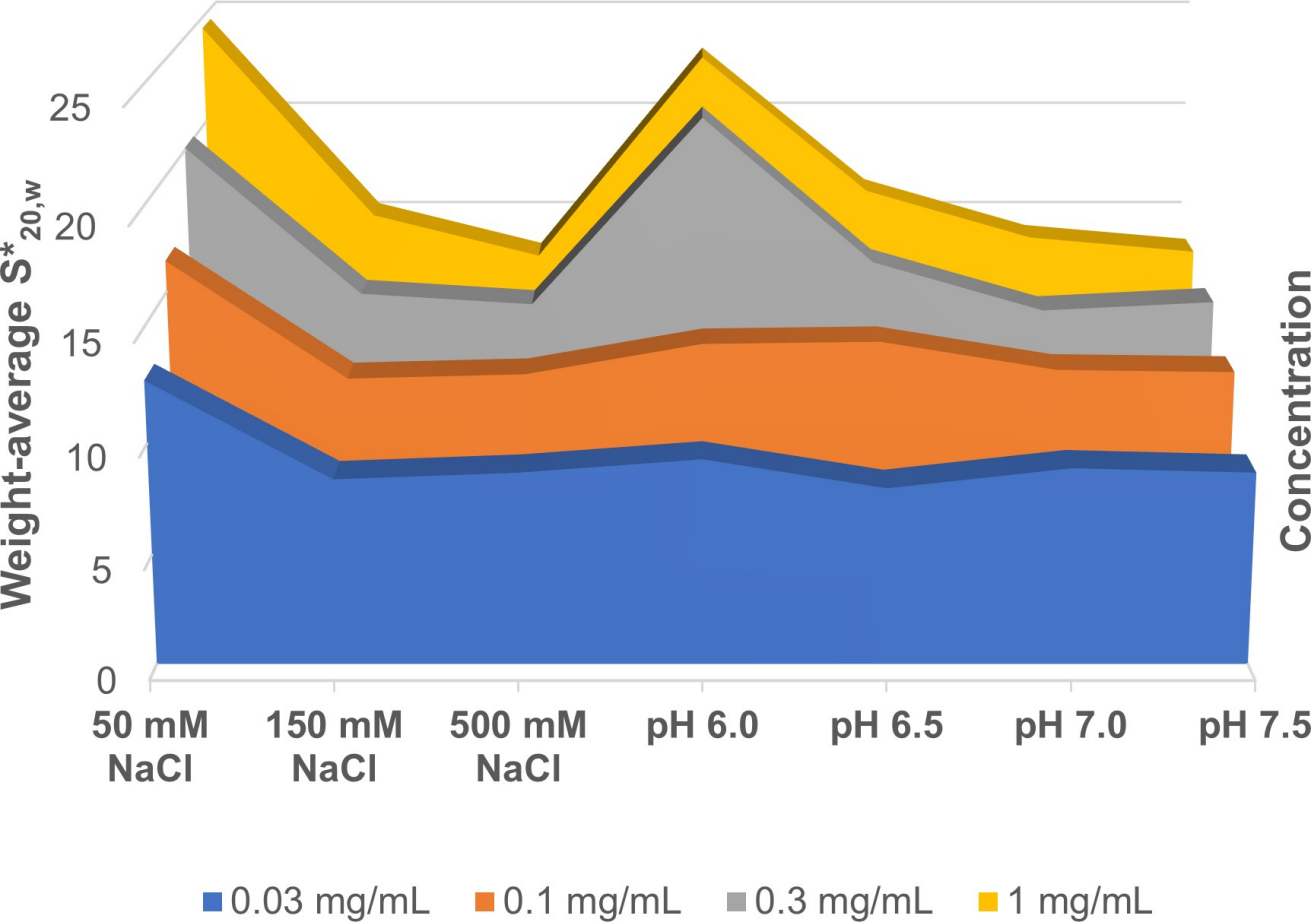
Graphical summary of self-association of hGALC at different concentration of NaCl or various pH levels. The weight-average S*_20,w_ decreased as the NaCl concentration or pH increased, indicating reduced self-association.

This indicated that electrostatic interactions were involved in self-association. Fitting was unsuccessful owing to the complex association. hGALC is localized in the lumen of lysosome which has a pH 4.5 – 5.0 [6, 14–16]. In this study, hGALC was precipitated at pH 4.5–5.0, and the size of aggregate extended to about 1000 S, indicating that hGALC has the highest tendency for oligomerization/high order aggregation at the physiological pH range. A graphical presentation clearly demonstrated that self-association of hGALC depended on the NaCl concentration and the pH (Fig 4).

### Sodium taurocholate lessened oligomerization of hGALC

During the course of optimization of the hGALC activity assay, it was observed consistently that the specific activity was lower in the presence of sodium taurocholate (NaTC). In contrast, polysorbate did not affect the activity. Therefore, the association behavior of hGALC was examined in the presence of NaTC. A sedimentation velocity dilution series was run on hGALC in 5 mM sodium phosphate and 150 mM NaCl, pH 6.0 with 1% NaTC. The rationale for the buffer condition was as follows: The activity assay buffer (50 mM citric acid and 100 mM sodium phosphate, pH 4.6) was known to cause significant aggregation. The enzymatic activity was most stable at pH 6.0. The NaCl content was within a physiological range and enough to mask non-ideality from charge effects.

**Fig 5:**
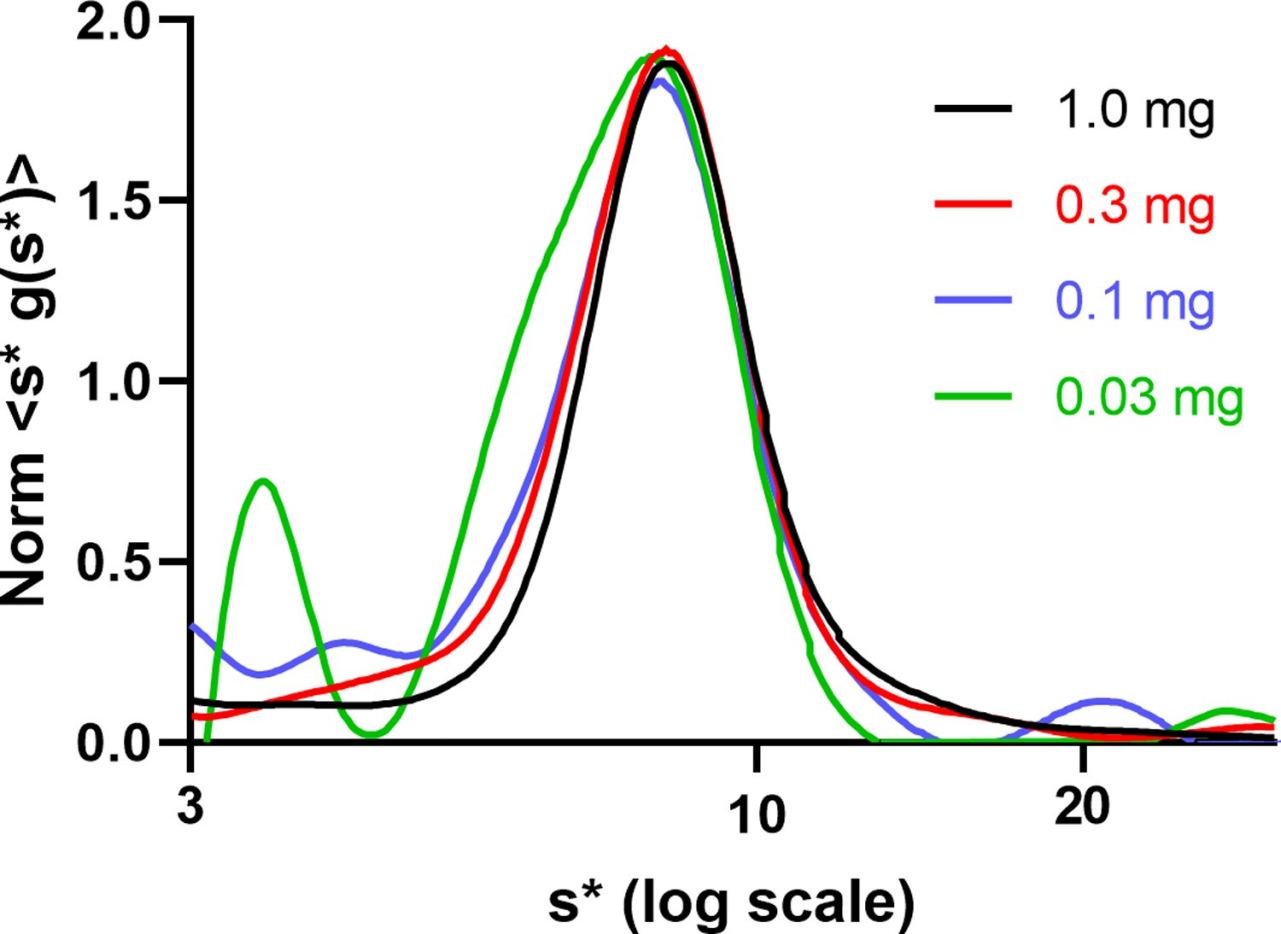
Sodium taurocholate decreased higher order oligomerization. Concentration-normalized s*g(s*) vs log_10_(s*) curves of hGALC at 1.0, 0.3, 0.1, and 0.03 mg/mL in 5 mM sodium phosphate (pH 6.0), 150 mM sodium chloride, and 1% sodium taurocholate (NaTC). It appeared that the peak position remained unchanged over a 33-fold concentration range.

The main peak position remained unchanged with the protein concentration (Fig 5) which contrasted with the result from in the absence of NaTC (Fig 1A).

This indicated that NaTC may have bound hGALC and significantly reduced high order oligomerization. One percent NaTC (18.6 mM) was above the critical micelle concentration (8–12 mM) [17], but no visible signal interference was observed. Fitting was attempted, but the result was inconclusive owing to uncertain parameters stemmed from the presence of NaTC.

## Discussion and conclusions

Ben-Yoseph et al reported that gel filtration chromatography of hGALC yielded four elution peaks, and fractions of high MW form (major peak) and low MW form (minor peak) produced an identical single band of MW 125 kDa on sodium dodecyl sulphate/polyacrylamide gel electrophoresis (SDS-PAGE) [1]. The two intermediate fractions (minor peaks) also revealed the same band on SDS-PAGE. In addition, the high MW form was partially converted into the intermediate and low MW forms after 48–72 hours at 4°C. Others also reported different MW forms of hGALC [2–3], but the solution behavior of hGALC remained unknown. Herein we report that the recombinant hGALC has a tendency for reversible self-association based on our AUC results. Moreover, self-association is dependent on the salt concentration and pH. The strength of self-association decreases as the NaCl concentration increases from 50 to 500 mM and the pH from 4.5 to 7.5 (Fig 4). The lumen of a lysosome where hGALC is localized has a Na^+^ concentration ranging from 20 to 140 mM and a pH ranging from 4.5 to 5.0 [14–16]. Therefore, the highest tendency for self-association/high order oligomerization occurs in the solution containing the physiological ionic strength and pH. NaTC notably suppresses self-association/oligomerization. The association is not formed by a disulfide crosslinking because both non-reduced and reduced with 50 mM DTT samples show an identical band of MW 80 kDa on SDS-PAGE (S1 Fig).

During the course of optimization of the activity test method, a range of pH and surfactants were evaluated. The hGALC specific activity was consistently low in the presence of NaTC but was not affected by polysorbate (S2 Fig). Note that NaTC suppressed self-association. The optimal condition for the activity was pH 4.6 (S3 Fig) where the highest self-association/aggregation was observed. The activity decreased as the pH increased from 4.6 to 6.0. Note that high order oligomerization decreased as the pH increased from 4.5 to 7.5. Ben-Yoseph et al reported that the high MW form showed significantly higher specific activity toward the natural substrate galactosylceramide compared to the artificial 4-methylumbelliferyl β-galactoside than the low MW form [1]. This indicated that oligomerization increased the enzymatic activity because the high MW form arose from reversible association of the low MW form. Taken together, oligomerization appears to be important. More studies are required to decipher what role oligomerization plays in the hGALC regulatory mechanism.

Lysosomal enzymes exist in low abundance and are hydrophobic. Recombinant technology enabled us to produce a large amount of protein for enzyme replacement therapy (ERT). ERT has been used for various lysosomal storage diseases, and its efficacy and safety have been confirmed by extensive clinical trials. By intravenous administration of purified recombinant enzymes, ERT provides the normal enzyme to the patient’s cells via an endocytic pathway and thus treats congenital enzyme deficiencies [18]. With respect to Krabbe disease, ERT improved the clinical phenotype in mouse models [19–21], but no human clinical trial has yet been reported. The hGALC properties described in this study may be of help for future ERT.

In conclusion, this is the first study describing the complex self-association/oligomerization of hGALC in solution. The recombinant hGALC employed in this study is similar to that purified from human tissues with regard to the propensity for self-association and being an acid hydrolase. Moreover, our results revealed that the highest tendency for self-association/oligomerization occurs under physiological conditions with respect to pH and ionic strength.

## Supporting information

S1 Fig hGALC oligomers are not disulfide cross-linked. hGALC both non-reduced and reduced with 50 mM DTT showed an identical single band on SDS-PAGE, indicating that the complex is not formed by a disulfide crosslinking.
(TIF)

S2 Fig The effect of surfactants on the hGALC enzymatic activity. Sodium taurocholate (NaTC) decreased the activity but polysorbate (PS) had no effect. A representative experiment out of 14 is shown.
(TIF)

S3 Fig The pH effect on the hGALC enzymatic activity. The optimal condition was determined to be pH 4.6. This demonstrated that the recombinant hGALC had similar enzymatic activity at the acidic pH as those purified from tissues [1–4].
(TIF)

S1 File Materials and methods for supporting information. (DOCX)

S2 File Minimal data set. (XLSX)
