# Supplementary figures and images for: Self-association of human beta-galactocerebrosidase: Dependence on pH, salt, and surfactant
Source: PLoS One. 2019 Dec 23;14(12):e0226618. doi: 10.1371/journal.pone.0226618 (PMC6927645; doi:10.1371/journal.pone.0226618)

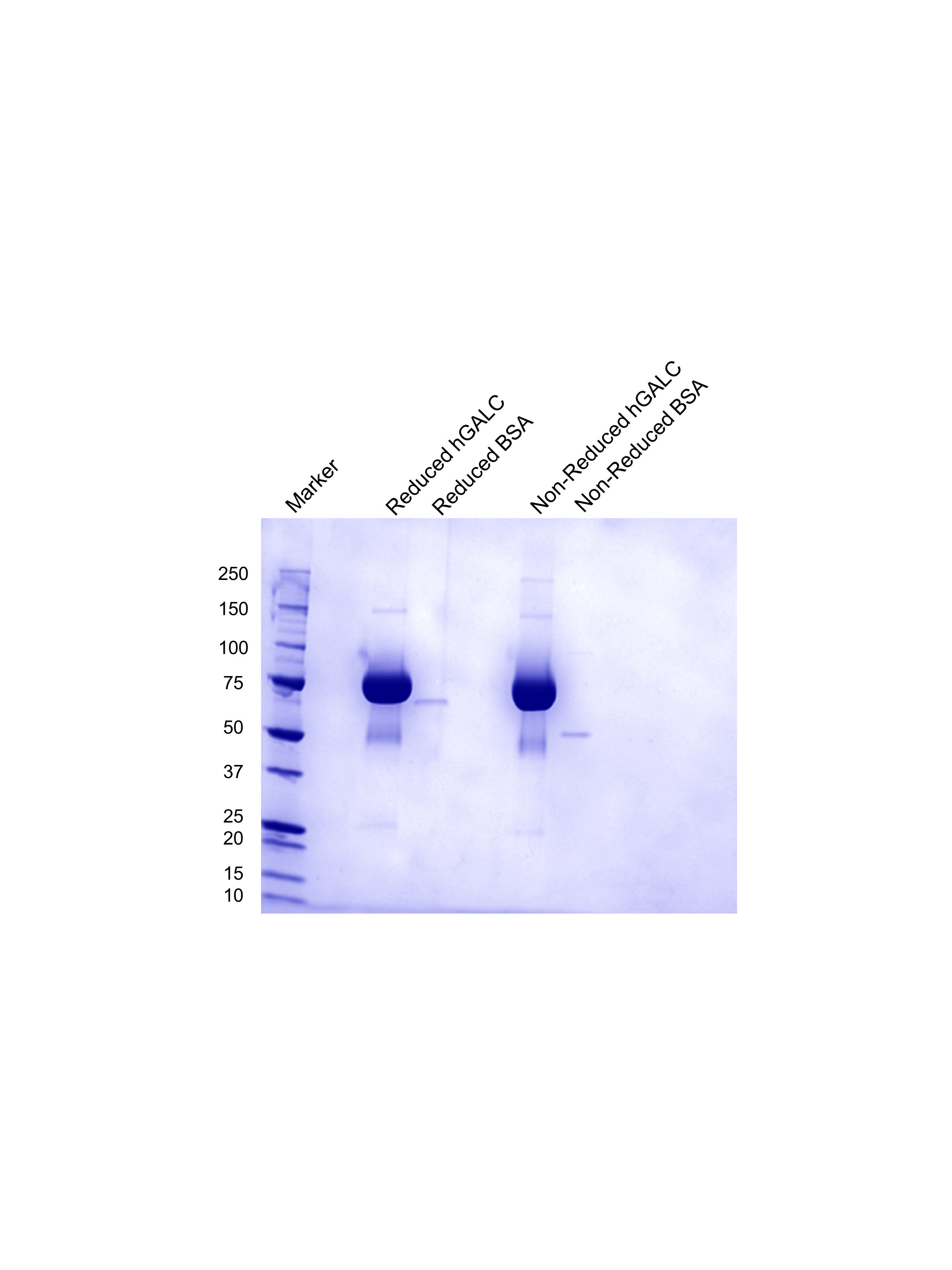

Supplement: S1 Fig — hGALC both non-reduced and reduced with 50 mM DTT showed an identical single band on SDS-PAGE, indicating that the complex is not formed by a disulfide crosslinking. (TIF) [file pone.0226618.s001.tif]

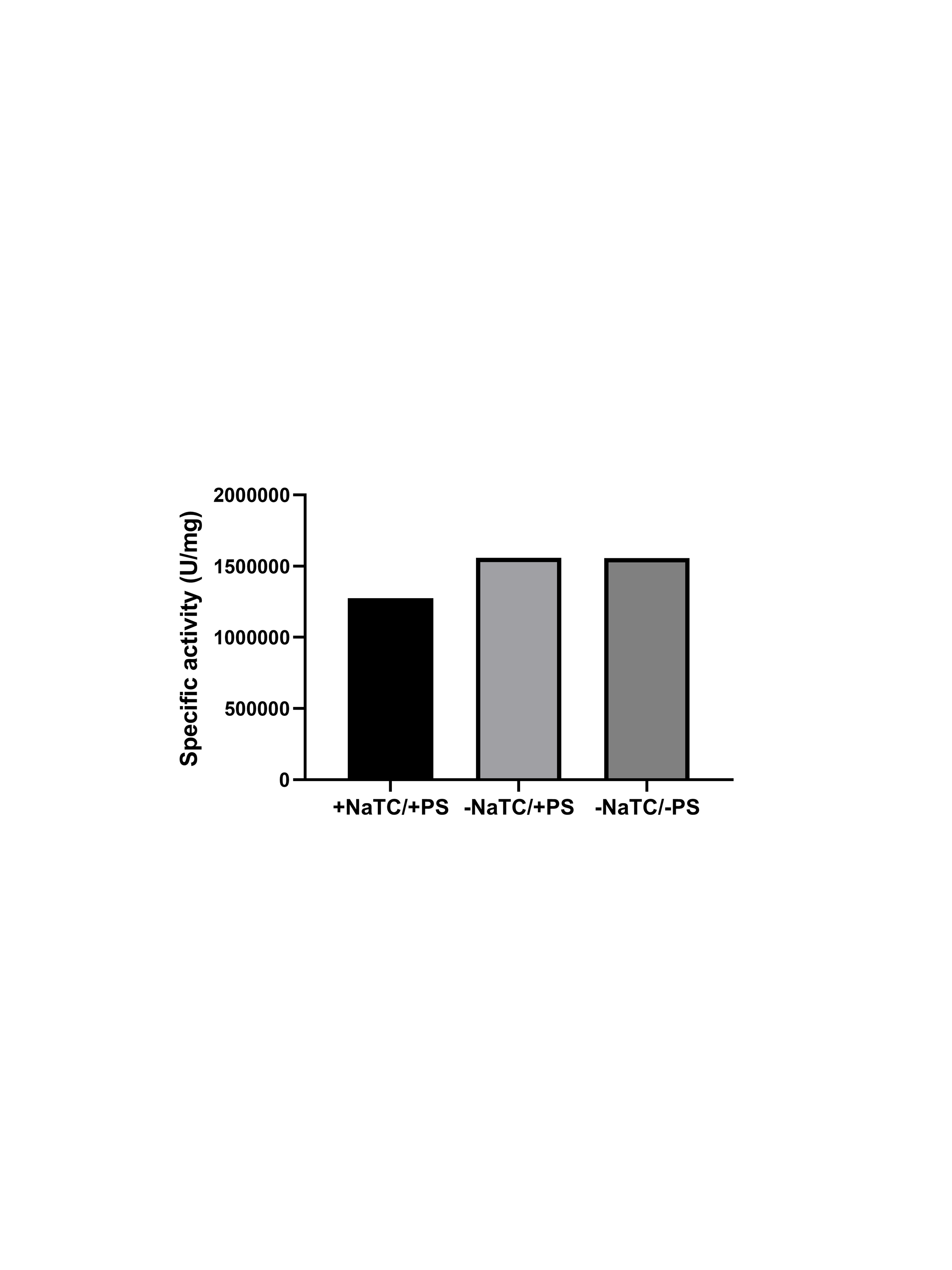

Supplement: S2 Fig — Sodium taurocholate (NaTC) decreased the activity but polysorbate (PS) had no effect. A representative experiment out of 14 is shown. (TIF) [file pone.0226618.s002.tif]

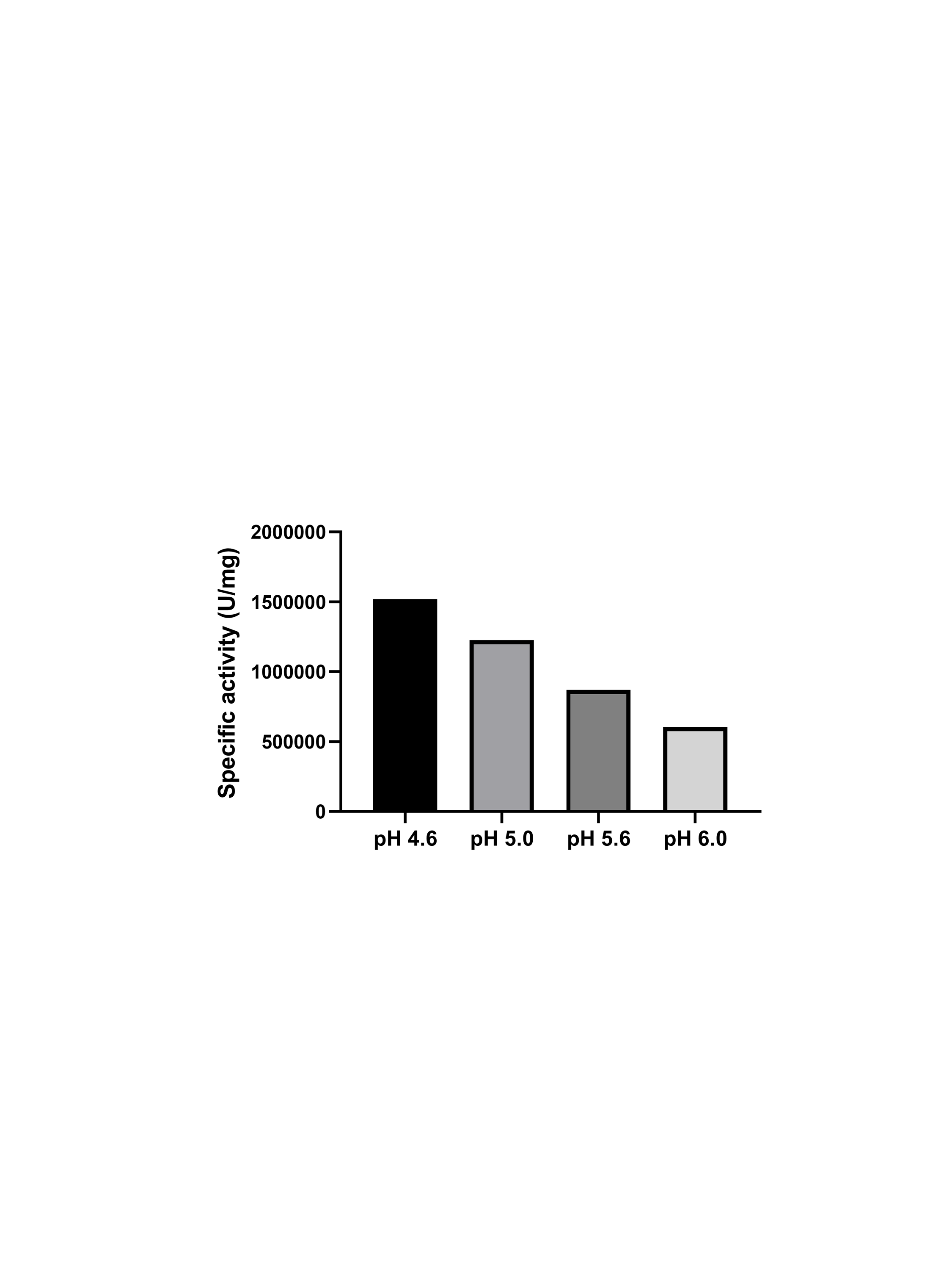

Supplement: S3 Fig — The optimal condition was determined to be pH 4.6. This demonstrated that the recombinant hGALC had similar enzymatic activity at the acidic pH as those purified from tissues [1–4]. (TIF) [file pone.0226618.s003.tif]
