# Supplementary material for: Self-association of human beta-galactocerebrosidase: Dependence on pH, salt, and surfactant
Source: PLoS One. 2019 Dec 23;14(12):e0226618. doi: 10.1371/journal.pone.0226618 (PMC6927645; doi:10.1371/journal.pone.0226618)
Supplement: S1 File — (DOCX) [file pone.0226618.s004.docx]

**Enzymatic activity assay**

Sodium taurocholate, 4-nitrophenyl β-D-galactopyranoside, and *p*-nitrophenol were purchased from Sigma-Aldrich. Polysorbate 20 and anhydrous sodium phosphate were from J. T. Baker‎. Bovine serum albumin (BSA) was from American Bioanalytics and citric acid from EMD. For the surfactants effect experiment, a synthetic substrate 4-nitrophenyl β-D-galactopyranoside was prepared in 50 mM citric acid and 100 mM sodium phosphate, pH 4.6 with or without 0.3% polysorbate 20/0.6% NaTC. hGALC was diluted in 50 mM citric acid, 100 mM sodium phosphate, 0.3% polysorbate 20, and 2 mg/mL BSA, pH 4.6. For the pH effect experiment, the substrate was prepared in 50 mM citric acid and 100 mM sodium phosphate, pH 4.6-6.0. hGALC was diluted in 50 mM citric acid, 100 mM sodium phosphate, and 1 mg/mL BSA, pH 4.6-6.0. A fresh *p*-nitrophenol solution was prepared on the day of the assay. Fifty mM *p*-nitrophenol was prepared in Milli-Q water with 0.02 M sodium hydroxide followed by further dilutions in the aforementioned enzyme diluent to achieve a standard curve ranging 0-40 nmol. After the mixture of 20 µL of the enzyme (25 µg/mL)/80 µL of the substrate (50 mM) was incubated for 1 hour at 37^o^C in a 96-well plate, the reaction was stopped by addition of 150 µL of the solution containing 333 mM glycine and 207 mM sodium carbonate, pH 10.7. Absorbance of the *p*-nitrophenol product and standard at 405 nm was measured using a SpectraMax plate reader with SoftMax Pro software version 5.4, and the amount of product was assessed from the standard curve.

**Reduced and non-reduced SDS-gel electrophoreses**

All chemicals were purchased from Invitrogen except for a protein marker and BSA purchased from Bio-Rad and Pierce, respectively. Reduced samples were prepared using Invitrogen NuPAGE™ Sample Reducing Agent (cat #NP0009). Novex™ Tris-Glycine SDS Sample Buffer (cat #LC2676), Novex™ Tris-Glycine SDS Running Buffer (cat #LC2675) and Novex™ 8 - 16% Tris-Glycine polyacrylamide gel (cat #EC6045) were employed for SDS-PAGE. Precision Plus Protein™ standards of Bio-Rad (cat #161-0363) was loaded on a gel along with samples to serve as molecular markers. All procedures were done according to manufacturers’ protocols.
